# Cu^+^/Ag^+^ Competition in Type I Copper Proteins (T1Cu)

**DOI:** 10.3390/biom13040681

**Published:** 2023-04-17

**Authors:** Nikoleta Kircheva, Silvia Angelova, Stefan Dobrev, Vladislava Petkova, Valya Nikolova, Todor Dudev

**Affiliations:** 1 Institute of Optical Materials and Technologies “Acad. J. Malinowski”, Bulgarian Academy of Sciences, 1113 Sofia, Bulgaria; nkircheva@iomt.bas.bg (N.K.); sea@iomt.bas.bg (S.A.); sdobrev@iomt.bas.bg (S.D.); vpetkova@iomt.bas.bg (V.P.); 2 Faculty of Chemistry and Pharmacy, Sofia University “St. Kliment Ohridski”, 1164 Sofia, Bulgaria; ohtvd@chem.uni-sofia.bg

**Keywords:** azurin, ceruloplasmin, DFT, plastocyanin, silver, Type I copper proteins

## Abstract

Due to the similarity in the basic coordination behavior of their mono-charged cations, silver biochemistry is known to be linked to that of copper in biological systems. Still, Cu^+^/^2+^ is an essential micronutrient in many organisms, while no known biological process requires silver. In human cells, copper regulation and trafficking is strictly controlled by complex systems including many cytosolic copper chaperones, whereas some bacteria exploit the so-called “blue copper” proteins. Therefore, evaluating the controlling factors of the competition between these two metal cations is of enormous interest. By employing the tools of computational chemistry, we aim to delineate the extent to which Ag^+^ might be able to compete with the endogenous copper in its Type I (T1Cu) proteins, and where and if, alternatively, it is handled uniquely. The effect of the surrounding media (dielectric constant) and the type, number, and composition of amino acid residues are taken into account when modelling the reactions in the present study. The obtained results clearly indicate the susceptibility of the T1Cu proteins to a silver attack due to the favorable composition and geometry of the metal-binding centers, along with the similarity between the Ag^+^/Cu^+^-containing structures. Furthermore, by exploring intriguing questions of both metals’ coordination chemistry, an important background for understanding the metabolism and biotransformation of silver in organisms is provided.

## 1. Introduction

Metals in biological systems perform critical functions, as they are indispensable key players in numerous biochemical processes such as signaling, redox-reactions, structure stabilization, and photosynthesis. Among the many (about 24) biogenic metal species that possess function in biological systems, copper is the third most abundant transition one coming only after iron and zinc [1,2]. Interestingly, Cu^+^/Cu^2+^ is not only an essential microelement crucial for proper functioning of proteins in organisms with oxidative metabolism, but it is also a poison, which stays under strict homeostatic regulation. The most preferred geometry for the metal-binding centers of proteins that coordinate the alternating copper ion is tetrahedral, described as the entatic or rack state, thus enhancing its reactivity by increasing its redox potential [3,4]. This intermediate structure corresponds well to the two observed electronic configurations: d^10^ for the reduced Cu^+^ with no specific geometric preferences and typical coordination number of 2 to 4, and d^9^ for the oxidized Cu^2+^ with preference toward square planar, square pyramidal, and distorted octahedral geometries and typical coordination number of 4 to 6. In respect to the metal center composition, the most abundant amino acid residues derive from histidine, cysteine and methionine.

An isoelectronic and close mimicry species to the Cu^+^ ion is the silver cation Ag^+^. Being a fellow neighbor in Group 11, silver exhibits some close physico-chemical characteristics (e.g., preference toward soft Lewis bases such as S- and N-, rather than O-containing), while in others the two cations differ substantially, including in an ionic radius of 100 pm vs. 60 for Ag^+^ vs. Cu^+^ [5], respectively, and oxidation potential (E°(Ag^+^/Ag^2+^) = −1.98 V vs. E°(Cu^+^/Cu^2+^) = −0.153 V), rendering the silver cation redox-inactive under the physiological conditions in biological systems [6]. Additionally, no known biochemical process requires silver as a cofactor, whereas copper is an essential microelement [7]. Therefore, a curious and thought-provoking theory of silver’s action as a cuprous contender has drawn scientific attention, especially in light of the well-defined antibacterial [8,9,10,11,12,13], antifungal [14,15], and antiviral [16] properties of Ag^+^. Although it is an element known and exploited by humanity since antiquity, silver’s mechanism of therapeutic action has only recently started to be unveiled. Our group has lately succeeded in clarifying the diverse action of the “alien” metal cation against Gram-positive and Gram-negative bacteria owing to its greater preference toward the constituents of the external bacterial envelope of the latter as compared to the former [17]. Interestingly, we have also shown that silver could compete with Ni^2+^ in some of its enzymes (e.g., urease), while others (mononuclear glyoxalase I and acireductone dioxygenase) stay well protected against attack from the foreign intruder [18]. The next step in disclosing silver’s antimicrobial activity appears to be delineating the controlling factors of Cu^+^/Ag^+^ rivalry in proteins. The urge to assess the ability of Ag^+^ to replace the cognate Cu^+^ in copper proteins is of great interest [19]. For the purposes of the current research, the T1 type copper proteins were chosen as a model system, since their affinity toward different metal species has been extensively studied through experimental techniques [6,19,20,21]. A systematic analysis at the atomic level of the factors that govern the metal selectivity in these structures, however, is lacking (to the best of our knowledge).

**Figure 1:**
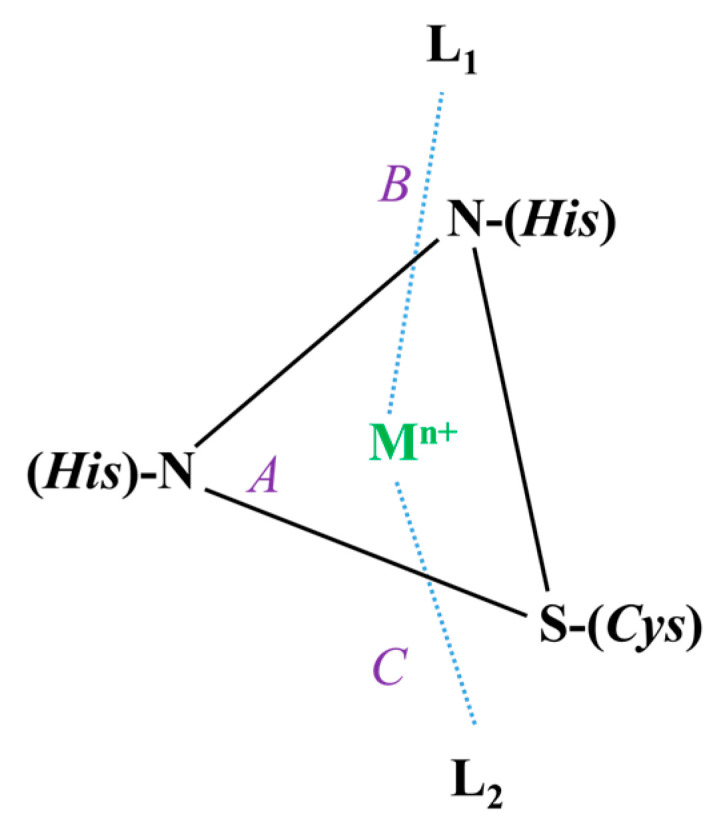
Geometrical composition of the metal-binding center in T1 copper proteins. A: the trigonal planar composition of the three amino acid residues (two histidines and one cysteine) characteristic for all T1Cu proteins; B axial ligand L_1_ (methionine in Class 1, and non-methionine in Class 2) distinguishing a distorted tetrahedron; C axial ligand L_2_ (absent in Classes 1 and 2, peptide-residue from glycine in Class 3, in combination with methionine as L_1_), which gives rise to a distorted trigonal bipyramidal geometry.

Most members of the T1 type copper-containing proteins function as electron-transferring shuttles between proteins; therefore, they are often referred to as “cupredoxins” in analogy to “ferredoxins” [22,23]. The metal binding center always involves strong coordination by two histidine- and one cysteine-derived amino acid residue positioned in a trigonal planar arrangement (Figure 1). Further classification follows the type of the axial L_1_ ligand: class 1 contains a methionine weakly bound to the metal, whereas L_1_ is a non-methionine in class 2 proteins. Both classes lack the fifth amino acid residue denoted as L_2_, which renders the resulting geometry of the metal binding center distorted tetrahedral. In class 3, L_1_ is methionine, while L_2_ comes from the O-atom in a peptide bond in glycine. This particular composition corresponds to a distorted trigonal bipyramidal geometry. Examples of the three classes taken into account in the current study are the plant plastocyanin [24], the human ceruloplasmin (Cp) [25], and the bacterial azurin [26] for Classes 1, 2, and 3, respectively. Furthermore, all T1Cu proteins possess some interesting features: they all have positive reduction potentials greater than 0.25 V and a strong absorption around 600 nm, attributed to the charge transferred from the cysteine thiolate sulfur to the metal. Hence, another name for this group arises: the blue copper proteins [27]. A small hyperfine splitting in the parallel region of EPR spectra is additionally observed [28].

The current in silico research aims at revealing key factors that govern the Cu^+^/Ag^+^ competition in biological systems, more specifically the T1Cu proteins. Although it is exclusively a theoretical approach to the problem, the presented study is solidly based on experimental data provided in literature. This particular combination of theory and experiment has previously been found reliable when the focus is on delineating governing factors of the utmost importance between native and alien metal species in biological systems [29,30,31,32,33,34]. In order to more thoroughly establish the extent to which Ag^+^ co-opts endogenous copper and where, alternatively, it is handled uniquely, we modeled the reactions of competition for binding constructs built by different types and numbers of amino acid residues, known to take part in the metal-binding centers in T1Cu proteins. Additionally, the effect of the surrounding medium is taken into account by employing the SMD [35] continuum dielectric computations, implemented in the thermodynamic cycle scheme. At the end, the metal-binding structures of three representatives of the corresponding Classes 1, 2, and 3 in their entirety were modeled and their affinity for the cuprous and silver ions assessed. Thus, the obtained results shed light on some aspects of silver’s intriguing mechanism of antibacterial action due to its ability to act as a cuprous contender.

## 2. Methods

### 2.1. DFT Methodology

The provided calculations were performed using the Gaussian 09 suite of programs [36]. Among three combinations of method/basis set—B3LYP/6-31+G(3d,p) with added empirical dispersion, M062X/6-31+G(d,p), and M062X/6-311++G(d,p), along with the pseudopotential SDD [37] for the heavier Ag^+^ in all cases—the last one proved to be the most suitable for the purposes of the current research. Full optimization of the studied structures provided results that to the greatest extent correspond to experimental data in the literature regarding the geometries of Cu^+^/Ag^+^ hydrated complexes and metal-binding centers in azurin, as well as reproduction of the hydration energy values of the metal species (See Section 2.2, Calibration and Validation of the Chosen Methodology). For each structure, full geometry optimization was carried out in the gas phase, followed by vibrational frequency analysis where no imaginary frequencies were observed indicating a local minimum of the potential energy surface. These results provided the essential electronic energies, E_elect_, the thermal energies, including the zero-point energy, E_th_, and entropy, S, necessary for the calculation of the Gibbs energy value, ∆G^1^, in the gas phase at room temperature T = 298 K and atmospheric pressure 1 atm according to the equation

∆G^1^ = ∆E_el_ + ∆E_th_ − T∆S (1)

where ΔE_el_, ΔE_th_, and ΔS are the corresponding differences between the products and the reactants of the Ag^+^ → Cu^+^ substitution reaction (See Section 2.2, Calibration and Validation of the Chosen Methodology; Reaction 1). Furthermore, the effect of the surrounding medium was accounted for by doing additional SMD [35] calculations. Single point calculations for each optimized structure were performed at three different dielectric constants: ε = 4, corresponding to a binding site, deeply buried in the protein; ε = 30, which is model of a partially solvent exposed metal binding center; ε = 78, water environment. Hence, the difference between the condensed-phase and the gas-phase energies of the respective constructs were used for the evaluation of the solvation energies, ΔG_solv_^ε^. These were employed for obtaining the energies for Ag^+^ → Cu^+^ substitution in accordance with the equation:

∆G^ε^ = ∆G^1^ + ∆∆G_solv_^ε^ (2)

where

∆∆G_solv_^ε^ = ∆G_solv_^ε^ (products) − ∆G_solv_^ε^ (reactants) (3)

A positive ∆G_solv_^ε^ value characterizes a copper-selective structure, whereas a negative ∆G_solv_^ε^ one suggests a silver-selective construct. Still, the aim of the present study is to provide reliable trends rather than reproduce specific values of ∆G_solv_^ε^.

The visualization of the obtained results was carried out through implementation of the PyMol Molecular Graphics System [38].

### 2.2. Calibration and Validation of the Chosen Methodology

**Figure 2:**
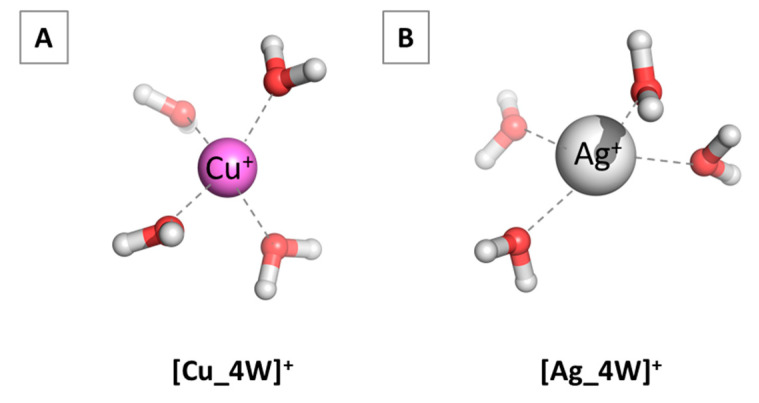
Theoretically modeled [Cu(H_2_O)_4_]^+^ (**A**) and [Ag(H_2_O)_4_]^+^ (**B**) complexes further denoted as [M_4W]^+^.

Although copper alternates between two states in Type I proteins, only the cuprous (Cu^+^) form is considered in the following research, since, namely, it is the closest to the abiogenic silver in respect to electronic configuration, ionic radius, and chemical preferences. The challenge with these cations is that they are “spectroscopically silent” [39,40], which necessitates their investigation with the powerful tools of the computational methodology. Both ions share the copper pathways for distribution in the body, although silver tends to be more selectively distributed among the organs, thus accumulating predominantly in sulfur-rich proteins building the liver [41]. In serum, Ag treatment leads to reduction of the total copper concentration and oxidase activity (associated with ceruloplasmin), although it does not affect the protein concentration [25,42,43]. These findings suggest that silver might indeed act as a copper competitor in T1 proteins. Therefore, we modeled the native/alien rivalry in the following manner:

[Ag(H_2_O)_4_]^+^ + [Cu-protein]^n^ → [Ag-protein]^n^ + [Cu(H_2_O)_4_]^+^ (R1)

where [Cu^+^/Ag^+^-protein] stands for the metal cation complexation to the studied amino acid residues/metal-binding center, whereas [Cu/Ag(H_2_O)_4_]^+^ is the hydrated cation coming from/released into the surrounding milieu. The [Cu/Ag(H_2_O)_4_]^+^ stays in tight correspondence to the experimental (spectroscopic and theoretical) data provided in the literature about the most probable configuration of the metal hydration shell [44,45,46,47,48,49,50,51]. Hence, the organization of the water molecules around Cu^+^/Ag^+^ is in tetrahedral (4 + 0) and distorted tetrahedral (2 + 2) fashion, correspondingly. The chosen combination of method/basis set (See Section 2.1, DFT Methodology) closely reproduces the angles and bond lengths in accordance to EXAFS and LAXS experiments: the average Cu-O bond is 2.14 Å [44,45] (2.15 Å in the structure used in the current study, Figure 2A), whereas in the [Ag(H_2_O)_4_]^+^ two H_2_O molecules are positioned at 2.32 Å and the remaining two at 2.54 Å away from the cation (2.33/2.47 Å in the structure used in the current study, Figure 2B).

Additional calibration of the thermodynamic parameters used herewith is provided through calculation of the hydration energy of the two metal cations. The combination M062X/6-311++(d,p) (SDD for the heavier Ag) provided a total energy of hydration for Cu^+^/Ag^+^ of −125.3/−99.2 kcal mol^−1^, while these values are correspondingly −125/−102 kcal mol^−1^ according to experiment [52]. Note that the other investigated combinations provided results not so close to the experiment: −127.4/−99.2 kcal mol^−1^ for M062X/6-31+(d,p), and −129.6/−99.8 kcal mol^−1^ (B3LYP/6-31+(3d,p) with added empirical dispersion). Furthermore, the most suitable combination for the current research was again proven to be the M062X/6-311++(d,p) (SDD for the heavier Ag), as it excelled the most in reproducing the bond lengths, and especially the difference between the Cu^+^/Ag^+^-containing metal center of azurin as compared to experimental data in the literature. The results in terms of differences are provided in the following Table 1, while the actual bond lengths are presented in Table S1 (Supplementary Materials).

The provided data were obtained for the model structures of the metal-binding center of azurin bound to Cu^+^/Ag^+^. The calculations were performed using widely accepted simplified models corresponding to the amino acid residues building the cavity. Hence, the side chains of His^0^, Met^0^, Cys^−^, and the backbone peptide group (BKB) were modeled as ethylimidazole, CH_3_CH_2_CH_2_SCH_3_, CH_3_CH_2_S^−^, and CH_3_CONHCH_3_, respectively. The charges of the amino acid models were considered in accordance to the pKa values of the amino acid side chains and the Lewis acidity of the bound mono-charged cations [53,54]. For clarity, the three-letter code for the amino acid residues will be hence applied in designating the modeled constructs, along with the abbreviation AAM (amino acid model) as a summary of the used ligands.

## 3. Results and Discussion

### 3.1. Evaluating the Cu^+^/Ag^+^ Competition in Model Systems with One and Two Identical Amino Acid Residues

**Figure 3:**
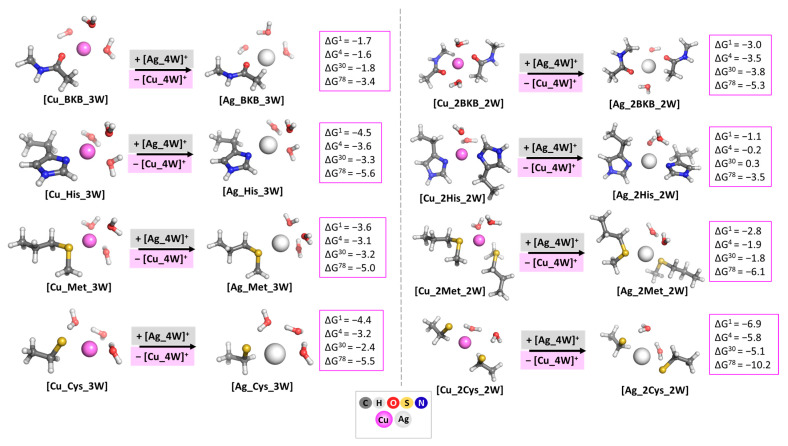
Gibbs energies of Ag^+^ → Cu^+^ substitution given in kcal mol^−1^ in four different environments (denoted as ∆G^1/4/30/78^) for M062X/6-311++G(d,p)/SDD-optimized [M_1/2AAM_3/2W] structures. The color scheme is further applied in all presented figures.

The first step in the current study is to evaluate the effect of each amino acid residue that takes part in building the metal-binding center in T1Cu proteins. Therefore, we modeled the Cu^+^/Ag^+^ competition in systems with 1 and 2 identical BKB, His, Met, and Cys residues. The optimized structures along with the obtained results are depicted in the following Figure 3.

For the Ag^+^ → Cu^+^ substitution in [M_1AAM_3W] structures, the free energies of metal exchange stay on negative ground, indicating the better complexation abilities of the Ag^+^ cation in these constructs. However, the obtained ∆G^ε^ values provide evidence for the strong preference of the alien silver cation toward N- and S-containing ligands as compared to the O-containing ones (about 2 kcal mol^−1^ higher energy gain for the substitution in His-, Met-, and Cys-containing constructs compared to the BKB-containing ones in all studied environments). Additionally, as the results for the Ag^+^ → Cu^+^ substitution in the [M_BKB_3W] structures are relatively close to zero and within the limits of the error of the method of 1 kcal mol^−1^, it could be concluded that the presence of one BKB-ligand in metal centers would provide better protection against outer attack. Taking into account the solvation effects, the calculations reveal that the polar aqueous medium promotes the competing properties of Ag^+^ to a greater extent, which should be attributed to the better solvation of the charged species, especially the product [Cu_4W]^+^ characterized with higher in absolute value hydration energy as compared to the reagent [Ag_4W]^+^.

Adding an identical ligand to the studied systems changes the outcome of the metal cation rivalry. The most interesting result comprises the Ag^+^ → Cu^+^ substitution in the [M_2His_2W] structure, where the corresponding ∆G^4/30^ values are −0.2 and 0.3 kcal mol^−1^, as compared to −3.6 and −3.3 kcal mol^−1^ for the mono-His constructs. Thus, the inclusion of a second histidine to the metal-binding center serves as a premise for a well-protected protein cavity. Notably, all T1Cu proteins contain two histidine residues in their active sites. On the other hand, the addition of a second Cys-ligand remarkably promotes silver’s competitiveness as the corresponding ∆G^ε^ values decrease substantially, especially in aqueous solution (∆G^78^ = −10.2 kcal mol^−1^). As an overall tendency, the addition of a second amino acid ligand increases the steric hindrance in the structure, followed by the expulsion of one or two water molecules to the second metal coordination shell, as in the case of 2Met/2His, and 2Cys, respectively. Hence, the valence angle between the two water molecules in the [Ag_2Met/2His_2W] complexes becomes much more strained as compared to its cuprous-containing counterparts: 63.5°/61° vs. 75°/68.5°. This tension is relieved in the [Ag_2BKB/2Cys_2W] structures, marked by a decrease in the ∆G^ε^ values. Therefore, the geometry of the metal-binding center and not only its amino acid composition plays a significant role in the Cu^+^/Ag^+^ competition, as suggested in Ref. [19].

### 3.2. Evaluating the Cu^+^/Ag^+^ Competition in Model Systems with Two Different Amino acid Residues

**Figure 4:**
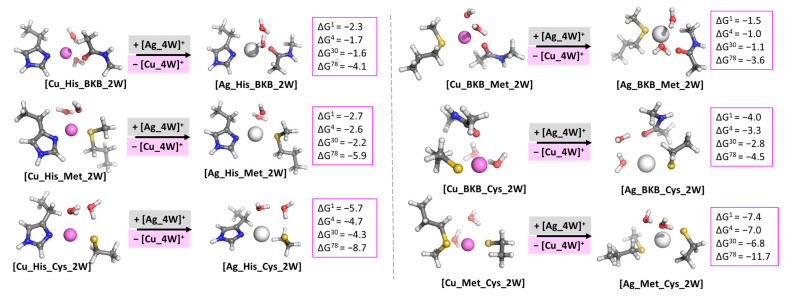
Gibbs energies of Ag^+^ → Cu^+^ substitution given in kcal mol^−1^ in four different environments (denoted as ∆G^1/4/30/78^) for M062X/6-311++G(d,p)/SDD-optimized [M_AAM_1__AAM_2__2W] structures. The applied color scheme has been previously presented in Figure 3.

As the metal-binding center in T1Cu proteins comprises a multitude of different amino acid residues, we investigated how the combination of two diverse models will affect the outcome of the Cu^+^/Ag^+^ rivalry. The obtained results are presented in the subsequent Figure 4.

The previously found tendencies are further observed for the Ag^+^ → Cu^+^ substitution in the [M_AAM_1__AAM_2__2W] structures. Although all the free energies are negative (meaning silver-selective-binding sites), the best protective combinations against an alien attack appear to be the Met/BKB and His/BKB, where the ∆G^4/30^ values vary between −1.0 and −1.7 kcal mol^−1^, which is close to the error limit of the method employed. In these Met/BKB and His/BKB silver-containing constructs, the strain in the valence angle between the two water molecules is again significant (66.9°/64°, respectively), resulting in a changed geometry, where the AAM ligands and one water molecule are positioned in a trigonal planar geometry with the second water molecule expulsed at 2.57 Å/2.69 Å, correspondingly. This trend is further preserved in the [Ag_His_Met_2W] and [Ag_BKB_Cys_2W] constructs, where the presence of the softer N- and S-containing Lewis bases promotes the silver attack to a greater extent, especially in the case of the charged Cys. In this regard, the most favorable combination for a Ag^+^ attack appears to be the Met/Cys, followed by the His/Cys one with corresponding ∆G^1/4/30/78^ values of −7.4/−7.0/−6.8/−11.7 and −5.7/−4.7/−4.3/−8.7 kcal mol^−1^. In these constructs, not only the tension is relieved by transporting one water molecule to the second hydration shell at 3.38 Å/3.65 Å, respectively, but the occurring complexation with the more favorable N- and S-donors undeniably benefits the Ag^+^ → Cu^+^ substitution. Although the overall charge of the complexes is zero (due to the presence of the charged Cys), the polar water environment promotes the ion–ion interaction between the metal cation and the Cys amino acid residue; hence, a significant increase in the absolute value of the ∆G energy in the aqueous solution is yielded.

### 3.3. Evaluating the Cu^+^/Ag^+^ Competition in Model Systems with Two Identical His and a Different Amino Acid Residue (Model of the Trigonal Planar Structure in T1Cu)

**Figure 5:**
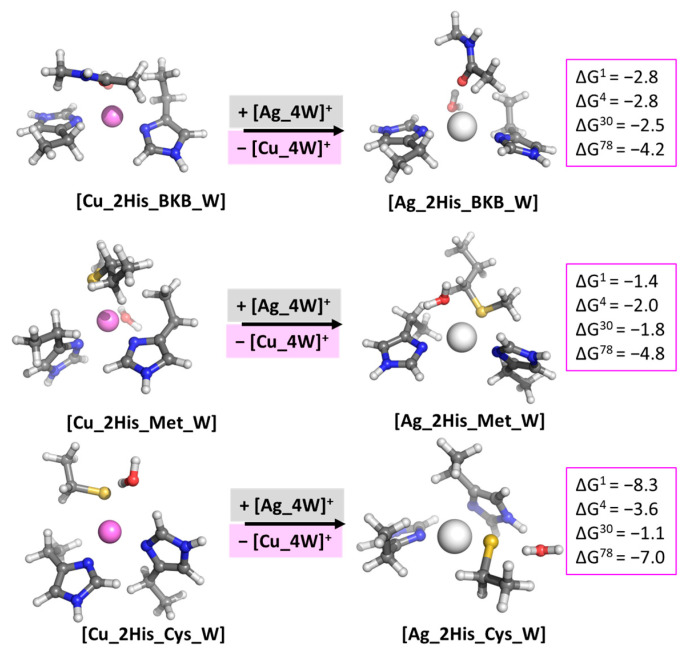
Gibbs energies of Ag^+^ → Cu^+^ substitution given in kcal.mol^−1^ in four different environments (denoted as ∆G^1/4/30/78^) for M062X/6-311++G(d,p)/SDD-optimized [M_2His_AAM_W] structures. The applied color scheme has been previously presented in Figure 3.

The amino acid residues in close proximity to the metal cation in the T1Cu proteins center are two His and one Cys. Therefore, it is of particular interest to evaluate the effect of the third AAM in these constructs. Hence, we modeled the Ag^+^ → Cu^+^ substitution in structures comprising two His and one different ligand (BKB, Met, and Cys) in order to delineate the resulting outcome in such complexes. The obtained results are presented in Figure 5. Note that the combination of three His amino acid residues is characteristic of Type III Cu proteins, which falls beyond the scope of the current study and is consequently omitted here.

The obtained results provide evidence that the presence of two His and one BKB/Met/Cys in the metal-binding center in T1Cu proteins does indeed promote silver’s attack, as all calculated ∆G^ε^ values are negative. Interestingly, the trigonal planar construction retained only in the [Ag_2His_BKB_W] structure favors the Ag^+^ → Cu^+^ substitution mostly in the non-polar environment (lowest ∆G^1/4/30^ values). This composition allows the metal cation to interact with the ligating groups to the greatest extent, as the bond lengths decrease: Ag-N(His) is 2.25 Å, Ag-O(BKB) is 2.60 Å, Ag-O(W) is 2.64 Å. An additional intermolecular hydrogen bond between the water molecule and the BKB-residue further stabilizes the structure. In the [Ag_2His_Met_W] construct, the amino acid residues are arranged in a distorted tetrahedron around the metal cation after the optimization. Thus, the Ag-AAM bonds elongate to 2.31 Å in Ag-N(His) and to 2.71 Å in Ag-S(Met), whereas the Ag-O(W) shortens to 2.62 Å. The most thought-provoking result appears to be the Cu^+^/Ag^+^ competition in the [M_2His_Cys_W] complex. The optimized cuprous-containing structure becomes trigonal planar, as is the composition in the real T1Cu center. Without the stronger interaction with an axial amino acid residue, however, the silver-containing construct becomes linear with Ag-S(Cys) at 2.43 Å and Ag-N(His_1_) at 2.23 Å, while His_2_ is displaced to the second coordination shell of the metal at 3.40 Å. Still, this loss of interaction is largely compensated for by the great preference of Ag^+^ toward the Cys-residue, resulting in strong complexation. The polar water environment, on the other hand, promotes the ion–ion interaction in the [Ag_2His_Cys_W] complex indicated by the lowest ∆G^78^ values: −7.0 in kcal mol^−1^ vs. −4.2/−4.8 in kcal mol^−1^ for the formation of [Ag_2His_BKB_W] and [Ag_2His_Met_W] structures, respectively.

### 3.4. Evaluating the Cu^+^/Ag^+^ Competition in Model Systems of the Metal-Binding Centers in Plastocyanin (Class 1), Ceruloplasmin (Class 2), and Azurin (Class 3)

**Figure 6:**
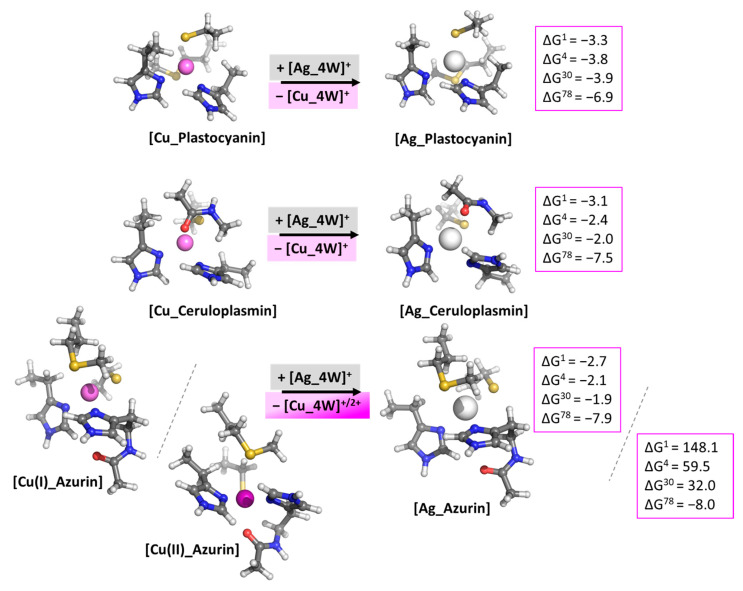
Gibbs energies of Ag^+^ → Cu^+^ substitution given in kcal.mol^−1^ in four different environments (denoted as ∆G^1/4/30/78^) for M062X/6-311++G(d,p)/SDD-optimized M_Plastocyanin (Class 1), M_Ceruloplasmin (Class 2), and M_Azurin (Class 3) structures. The applied color scheme has been previously presented in Figure 3. The results for the Ag^+^ → Cu^2+^ substitution in the metal-binding center of Azurin have also been obtained and presented, along with the fully optimized Cu^2+^-bound structure modeled in accordance with the available X-ray structure (PDB Entry: 1E5Y).

The use of simplified models, but that contain metal-ligating groups in pair with the experimental amino acid side chains, gives a solid premise for the understanding of the basic principles governing the metal ion competition in biological systems. The real centers, however, are of no less interest when evaluating the main factors that govern the Ag^+^ → Cu^+^ substitution. Consequently, we modeled the rivalry between the cations in real metal-binding centers of the three classes of T1Cu proteins, namely Plastocyanin (Class 1), Ceruloplasmin (Class 2), and Azurin (Class 3). The obtained results are given in Figure 6.

The evaluated Gibbs energies stay firmly on negative ground, indicating an Ag^+^ favorable metal-binding center in all three classes of T1Cu proteins. Notably, these conclusions are strongly supported by experimental data in literature [6,19,55]. Although the constructs differ in composition of the axial ligand/ligands, the trigonal planar composition of the amino acid residues ligating the metal cation in close proximity affects the outcome of the Ag^+^ → Cu^+^ substitution to the greatest extent. Thus, regardless of the nature of the complementing fourth/fifth ligand, the abiogenic silver cation appears capable of competing with the native cuprous ion for binding in the T1Cu proteins, as the calculated ∆G^ε^ values do not differ substantially and are affected mostly by the presence of the softer Lewis base Met at axial position in the less polar environment (compare results for the Ag^+^ → Cu^+^ substitution in plastocyanin and ceruloplasmin/azurin in ε = 1/4/30). In the polar aqueous medium, the calculated results stay close and give a solid premise for the widespread belief that silver is indeed a capable contender to the native cuprous ion in some of its enzymes (particularly in the T1Cu proteins), as the ∆G^78^ values vary from −6.9 to −7.9 kcal mol^−1^ (see Figure 6). Finally, we modeled the metal competition reaction only in the case of the cupric metal-binding center of azurin according to the known PDB entry 1E5Y [26], as the studied protein includes the alternating form of copper. Note that in this case ∆Gs are positive in the non-polar medium of 1 to 30, but reverse sign when the environment changes to the polar aqueous solution (∆G^1/4/30^ = 148.1/59.5/32.0 as compared to the ∆G^78^ = −8.0 kcal mol^−1^). By applying the Charge Model 5 (CM5) atomic charges [56] derived from the Hirshfeld population analysis scheme [57], we further revealed that the charge transferred from the ligands to silver is enhanced in comparison to the cuprous ion (1.12 e^−^ vs. 1.08 e^−^), but decreased juxtaposed to the cupric form (1.12 e^−^ vs. 1.82 e^−^). These results clearly indicate the ability of the abiogenic cation to compete with the native one, but only in the cases of its cuprous form, or when the active site bound to the cupric form is positioned in a solvent-exposed medium. Consequently, the silver cation may affect the attacked enzymes through two diverse pathways: (1) by substituting the redox-active copper ion (most probably in its cuprous state) in the active site, it renders the enzyme inactive; (2) by releasing the Cu^+^/Cu^2+^ into the surrounding medium, Ag^+^ increases the possibility of “poisoning” the intracellular milieu. However, due to the high physiological concentration of copper in the body (about 100 mg in a body of ~70 kg [19]), only the proteins that are the most susceptible to a silver attack should be considered as the main targets of the alien cation. The current study suggests that, indeed, the T1Cu represents such an example. Further investigation is strongly required in order to delineate the susceptibility of other types of copper proteins to a Ag^+^ → Cu^+^ substitution.

## 4. Conclusions

The presented results herewith provide theoretical evidence of silver’s ability to act as a cuprous contender in T1Cu proteins. By implementing a well-studied methodology, we disclosed some principles of the utmost importance that govern the Ag^+^ → Cu^+^ substitution. First, the nature of the amino acid residue that builds the metal-binding center plays a crucial role, as silver, being a soft Lewis acid, preferentially binds soft N- and S-containing ligands. Furthermore, the geometry of the protein cavity appears essential, as suggested in Ref. [19]. Hence, the trigonal planar composition in close proximity to the metal cation favors the complexation of the abiogenic silver. The surrounding environment characterized by a specific dielectric constant is also of indisputable significance, since the polar aqueous solution solvates better the charged species in the reaction and also promotes the ion–ion interaction between the cationic metal and the negative amino acid residue (e.g., Cys). Consequently, silver successfully competes with the native cuprous ion for binding in all three studied centers representative for the T1Cu proteins but substitutes the cupric form only in the case of a solvent-exposed azurin center. The provided results shed light on some aspects of silver’s biochemistry in biological systems. However, the intriguing questions of the outcome of the Ag^+^/Cu^+^ competition in other types of proteins (T2 and T3), as well as in the Cys-rich metallothioneins and copper chaperones remain unaddressed and await answers.

## Figures and Tables

**Table 1:** Bond lengths used for justification of the chosen computational protocol. The experimental data are taken form the Cu^+^/Ag^+^—Azurin structures, corresponding PDB Entries: 1JZG [21] and 3UGE [6].

Bond Length in Å/ Method		M^+^-N (His_1_)	M^+^-N (His_2_)	M^+^-S (Cys)	M^+^-S (Met)	M^+^-O (BKB)
Experiment	∆(Ag^+^-Cu^+^)	0.33	0.29	0.14	0.22	0.22
B3LYP/6-31+G(3d,p)-D3	∆(Ag^+^-Cu^+^)	0.07	0.84	0.18	0.00	0.55
M062X/6-31+G(d,p)	∆(Ag^+^-Cu^+^)	0.35	0.19	0.16	0.33	0.07
M062X/6-311++(d,p)	∆(Ag^+^-Cu^+^)	0.33	0.19	0.15	0.30	0.04

## Data Availability

The data presented in this study are available on request from the corresponding author.

## Supplementary Materials

The following supporting information can be downloaded at: https://www.mdpi.com/article/10.3390/biom13040681/s1, Table S1: Bond lengths used for justification of the chosen computational protocol. The experimental data are taken from the Cu^+^/Ag^+^—Azurin structures, corresponding PDB Entries: 1JZG [21] and 3UGE [6].
