# Supplementary material for: Cu+/Ag+ Competition in Type I Copper Proteins (T1Cu)
Source: Biomolecules. 2023 Apr 17;13(4):681. doi: 10.3390/biom13040681 (PMC10136078; doi:10.3390/biom13040681)
Supplement: Supplementary file 1 [file biomolecules-13-00681-s001.zip › biomolecules-2346767-supplementary.pdf]

biomolecules

MDPI

Article

Cu^{+}/Ag^{+} Competition in Type I Copper Proteins (T1Cu)

Nikoleta Kircheva [1], Silvia Angelova [1], Stefan Dobrev [1], Vladislava Petkova [1], Valya Nikolova [2] and Todor Dudev [2,*]

[1] Institute of Optical Materials and Technologies "Acad. J. Malinowski", Bulgarian Academy of Sciences, 1113 Sofia, Bulgaria; nkircheva@iomt.bas.bg (N.K.); sea@iomt.bas.bg (S.A.); sdobrev@iomt.bas.bg (S.D.); vpetkova@iomt.bas.bg (V.P.)

[2] Faculty of Chemistry and Pharmacy, Sofia University "St. Kliment Ohridski", 1164 Sofia, Bulgaria; ohtvd@chem.uni-sofia.bg

* Correspondence: t.dudev@chem.uni-sofia.bg

Table S1. Bond lengths used for justification of the chosen computational protocol. The experimental data are taken form the Cu^{+}/Ag^{+} – Azurin structures, corresponding PDB Entries: 1JZG [21] and 3UGE [6].

**Bond Length in Å/ Method**		**M^{+}-N(His$_1$)**	**M^{+}-N(His$_2$)**	**M^{+}-S(Cys)**	**M^{+}-S(Met)**	**M^{+}-O(BKB)**
Experiment	Cu^{+}	2.12	2.06	2.23	3.31	2.67
	Ag^{+}	2.45	2.35	2.37	3.09	2.89
	**Δ(Ag^{+}-Cu^{+})**	**0.33**	**0.29**	**0.14**	**0.22**	**0.22**
B3LYP/6-31+G(3d,p)-D3	Cu^{+}	2.12	2.01	2.19	3.55	4.24
	Ag^{+}	2.19	2.85	2.37	3.55	4.79
	**Δ(Ag^{+}-Cu^{+})**	**0.07**	**0.84**	**0.18**	**0.00**	**0.55**
M062X/6-31+G(d,p)	Cu^{+}	2.22	2.12	2.32	2.76	4.37
	Ag^{+}	2.57	2.31	2.48	3.09	4.44
	**Δ(Ag^{+}-Cu^{+})**	**0.35**	**0.19**	**0.16**	**0.33**	**0.07**
M062X/6-311++(d,p)	Cu^{+}	2.23	2.12	2.33	2.79	4.38
	Ag^{+}	2.56	2.31	2.48	3.09	4.42
	Δ(Ag^{+}-Cu^{+})	0.33	0.19	0.15	0.30	0.04
